# Research on bacterial community characteristics of traditional fermented yak milk in the Tibetan Plateau based on high-throughput sequencing

**DOI:** 10.7717/peerj.14733

**Published:** 2023-01-25

**Authors:** Shifang Wu, Xiaoli Yang, Haina Gao, Chengrui Shi, Longlin Wang, Deyuan Lu, Yiheng Li, Jinliang Zhang, Weibing Zhang, Pengcheng Wen

**Affiliations:** 1 College of Food Science and Engineering, Gansu Agricultural University, Lanzhou, Gansu, China; 2 Gansu Institute of Business and Technology, Lanzhou, Gansu, China; 3 School of Food and Health, Beijing Technology & Business University, Beijing, Beijing, China

**Keywords:** Plateau Tibetan area, Traditional fermented yak milk, High-throughput sequencing, Bacterial diversity

## Abstract

**Background:**

The Tibetan Plateau has an abundance of yak milk resources. The complex microbiota found in traditional fermented yak milk produced and sold by local Tibetans endows the yak milk with unique quality characteristics such as tissue morphology, flavor, and function. However, the diversity of bacterial flora in traditional fermented yak milk have not been elucidated.

**Methods:**

In this study, 15 samples of fermented yak milk were collected for 16S rRNA high-throughput sequencing to analyze the bacterial community composition and function.

**Results:**

After filtering for quality, 792,642 high-quality sequences were obtained, and 13 kinds of different phyla and 82 kinds of different genera were identified, of which the phylum *Firmicutes* (98.94%) was the dominant phylum, *Lactobacillus* (64.73%) and *Streptococcus* (28.48%) were identified as the dominant genus, in addition, the bacterial community richness and diversity were higher in Manang Village, followed by Bola Village. Bacterial community richness and diversity in Huage Village were relatively low. Based on the Kyoto Encyclopedia of Genes and Genomes (KEGG) functional classification, the microorganisms in traditional fermented yak milk have rich metabolic functions (77.60%). These findings suggest that a large number of bacteria in traditional fermented yak milk contain abundant metabolic genes and can carry out a variety of growth and metabolic activities. This study established a theoretical foundation for further exploring the microbial flora of traditional fermented yak milk in Gannan.

## Introduction

Yaks are members of the subfamily Bovinae and have a strong tolerance to the alpine environment. They live in the high-altitude Tibetan Plateau all year round (Xin et al., 2019). Yak milk, rich in protein, essential minerals, and polyunsaturated fatty acids, provides Tibetan herders with a significant economic source (Kandeepan & Sangma, 2010). One of China’s eight Zang communities (of Tibetan nationality) is home to the Gan-Nan Tibetan autonomous prefecture. This region has an average elevation of more than 3,000 m. It has plenty of milk resources, particularly yak milk (Bao et al., 2012a; Xin et al., 2021). Yak yogurt is made from yak milk and has a high nutritional value, fermented by a naturally selected population of lactic acid bacteria and yeast. The microbial community in yogurt is also highly diverse because of the unique climate, altitude, and technology of the Gannan (Jun et al., 2013; Wang, She & Liu, 2020).

Fermented yak milk in Gannan Tibetan region has a long history with thousands of years of inheritance, produced by traditional fermentation methods by Tibetan herdsmen (Yu et al., 2018). The traditional fermented yak milk is made similarly in a particular traditional container. First, milking is usually done on open pastures, and the fresh yak milk is pooled into a larger container, then the yak milk will be filtered through a sieve or a clean white cloth, when the raw cow’s milk was boiled and cooled, followed by the step of skimming of lipids. An enrichment starter was then inoculated in the milk by back-slopping. Simply seal it and put it outside; the mixture was allowed to ferment overnight at ambient temperature and get sour yak milk (Liu et al., 2015b). Traditional fermented yak milk is the most popular dairy product made by local herdsmen and sold to nearby areas.

It has been previously noted that fermented yak milk contains a variety of microbes (Bai et al., 2010; Bao et al., 2012b; Liu et al., 2012; Liu et al., 2015b). At the genus level, *Firmicutes* are dominated by *Lactobacillus*, *Streptococcus*, *Lactococcus*, and *Leuconostoc*. At the species level, *Streptococcus thermophilus* and *Lactococcus lactis* were also present in some samples, along with many *Lactobacillus* species, including *Lactobacillus delbrueckii ssp*. *bulgaricus*, *Lactobacillus helveticus*, *Lactobacillus fermentum*, and *Lactobacillus Plantarum*. Although informative, previous research has primarily focused on identifying the microbiota composition, and additional investigation is needed beyond the relative abundance of different genera. In addition, the vast Tibetan Plateau has abundant microbial resource pool, so it is critical to investigate the types and properties of microbial resources to clarify and apply their functions, particularly in Gannan fermented yak milk (Guo et al., 2020).

High-throughput sequencing technology has been widely applied to study microbial flora structure and diversity in fermented dairy products due to its advantages of low cost, high sequencing quality, and a more objective and comprehensive understanding of the microbial structure (Quigley et al., 2013; Soto et al., 2017). Many studies have shown that high-throughput sequencing is an excellent tool for investigating the structure of microbial communities. Shangpliang et al. (2018) used Illumina MiSeq high-throughput sequencing technology to examine bacterial community structure in 54 traditional fermented dairy products and discovered that the bacterial flora diversity of different types of fermented dairy products differed. Sessou, Santosh & Ngangyola (2019) studied the diversity of yeast in handmade cheese samples from Benin and 20 fermented milk samples from Benin and Niger using high-throughput sequencing techniques and discovered significant differences in yeast composition between the two countries. Cao et al. (2021) used high-throughput sequencing of the 16S rRNA V3–V4 region to examine the components, abundance, and the diversity of 48 bacterial populations sampled from eight different typical sites in a dairy farm, by providing a comprehensive understanding of bacterial diversity and composition along milking in dairy farms, systematically analyzed the effect of milking behavior and environment on the quality of raw milk in dairy farm.

The microbial species in traditional fermented yak milk are complex and changeable, the fermentation conditions and processes are not standardized, and the quality of the products is uneven. Therefore, to further develop traditional fermented yak milk, the composition and characteristics of the microorganisms in the products need to be clarified first. At present, there are few research reports on the diversity of bacterial flora in traditional fermented yak milk in the Gannan region of Gansu Province, so it is necessary to fully understand the diversity of bacterial flora in traditional fermented yak milk in the area. Therefore, the primary aims of this study were to characterize traditional fermented yak milk collected from Gannan using Illumina MiSeq high-throughput sequencing technology, as well as to conduct a comparative analysis of different areas and different sources of bacteria, as well as the community structure of fermented yak milk. It is of great significance to develop excellent microbial resources and screen probiotics in the later stage meanwhile, it provides the theoretical basis for the safe production of local dairy products.

## Materials and Methods

### Samples of fermented yak milk

Fifteen traditional fermented yak milk samples were obtained from Xiahe County (3,500 m) in Gannan Tibetan Autonomous Prefecture, Gansu Province, collected in July 2021 from different farmers in four villages. Including six samples (HG11, HG12, HG13, HG21, HG22, HG23) from the Huage Village, three samples (MN1, MN2, MN3) from the Manang Village, three samples (BL1, BL2, BL3) from the Bola Village, and three samples (NML1, NML 2, NML3) from the Nimalong Village. Among them, HG11, HG12 and HG13 belong to the sale samples, and the others belong to the non-sale samples. Sale samples mean that there are specialized cooperatives that produce fermented yogurt or other dairy products and sell them to other people in the village or other places, while non-sale samples mean that herders produce and consume them themselves. All the traditional fermented yak milk samples were processed similarly through the natural fermentation of yak milk.

All of these fermented yak milks were aseptically collected and stored into an ice box before being transported to the laboratory within 12 h, then frozen at −80 °C until DNA extraction.

### DNA extraction

DNA samples were extracted referring to Liu et al. (2015c) and using the OMEGA Soil DNA Kit (M5635-02) (Omega Bio-Tek, Norcross, GA, USA), as directed by the manufacturer. A total of 1.2% agarose gel electrophoresis and a NanoDrop NC2000 spectrophotometer (Thermo Fisher Scientific, Waltham, MA, USA) with an optical density at 260 nm/280 nm ratio were used to assess the quality of the extracted DNAs. For further analysis, the samples of extracted DNA were stored at −20 °C.

### 16S rRNA gene amplicon sequencing

The Pfu high-fidelity DNA polymerase was used for PCR amplification, the V3–V4 region of standard bacterial 16S rRNA was amplified using total DNA as a template, the forward primer 338F (5′-ACTCCTACGGGAGGCAGCA-3′), and the reverse primer 806R (5′-GGACTACHVGGGTWTCTAAT-3′). Amplification system (25 μL): 5×reaction buffer 5 μL, 5×GC buffer 5 μL, dNTP (2.5 mM) 2 μL, Forward primer (10 uM) 1 μL, Reverse primer (10 uM) 1 μL, DNA Template 2 μL, dd H2O (8.75 μL), Q5 DNA Polymerase 0.25 μL. Amplification parameter: Initial denaturation 98 °C (2 min), Denaturation 98 °C (15 s), annealing 55 °C (30 s), extension 72 °C (30 s), final extension 72 °C (5 min), 10 °C Hold 25–30 Cycles. After PCR amplification completed, PCR amplicons were purified with Vazyme VAHTSTM DNA Clean Beads and quantified with the Quant-iT PicoGreen dsDNA Assay Kit (Invitrogen, Carlsbad, CA, USA). Following the individual quantification step, pair-end 2 × 250 bp sequencing was performed at Suzhou PANOMIX Biomedical Tech Co. LTD using the Illumina NovaSeq platform with the NovaSeq 6000 SP Reagent Kit (500 cycles).

### Microbiome bioinformatics analysis

After PCR amplification completed, microbiome bioinformatics was examined with QIIME2 2019.4 (Bolyen et al., 2018). The forward and backward reads obtained from double-end sequencing were assembled in pairs, and the sequences containing N in the assembled results were filtered out and those with a sequence length greater than 200 bp were retained. The sequences obtained were then filtered to remove chimeric sequences. VSEARCH (1.9.6; https://zenodo.org/record/44512#.Y7xRZ-zMLDQ) was used for sequence clustering, and the 97% sequential similarity levels were used as the classification threshold to cluster into operational taxonomic units (OTU) (Fu et al., 2015).

### Bioinformatics and statistical analysis

The QIIME2 and R packages (v3.2.0) were primarily used to analyze sequence data. The Alpha diversity level of each sample was evaluated based on the distribution of ASV (amplicon sequence variants)/OTU, and the appropriate sequencing depth was reflected by rarefaction curves (Ji et al., 2017). The unweighted pair-group method with arithmetic mean (UPGMA) based on the unweighted distance matrix was clustered using the QIIME software. The beta diversity was analyzed using Bray-Curtis metrics and visualized using principal coordinate analysis (PCoA). PICRUSt2 (Gavin et al., 2019) predicted microbial functions based on Kyoto Encyclopedia of Genes and Genomes (KEGG) databases (Zhang et al., 2017a).

## Results

### PCR amplification basics

**Figure 1:**
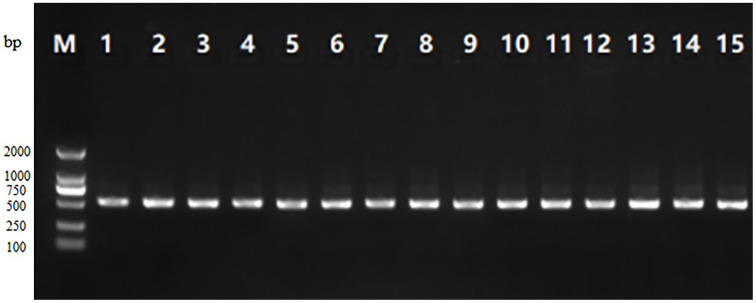
Gel electrophoresis of the PCR amplification product of V3-V4 region of 16S rRNA from 15 traditional fermented yak milk samples.

As can be seen from Fig. 1, the PCR amplification product gel electrophoresis of 15 samples showed clear, obvious bands at about 500 bp, and there was no obvious tailing phenomenon, indicating that the target fragment amplification was successful.

### Bioinformatics analysis (Sample sequence number characteristics)

**Figure 2:**
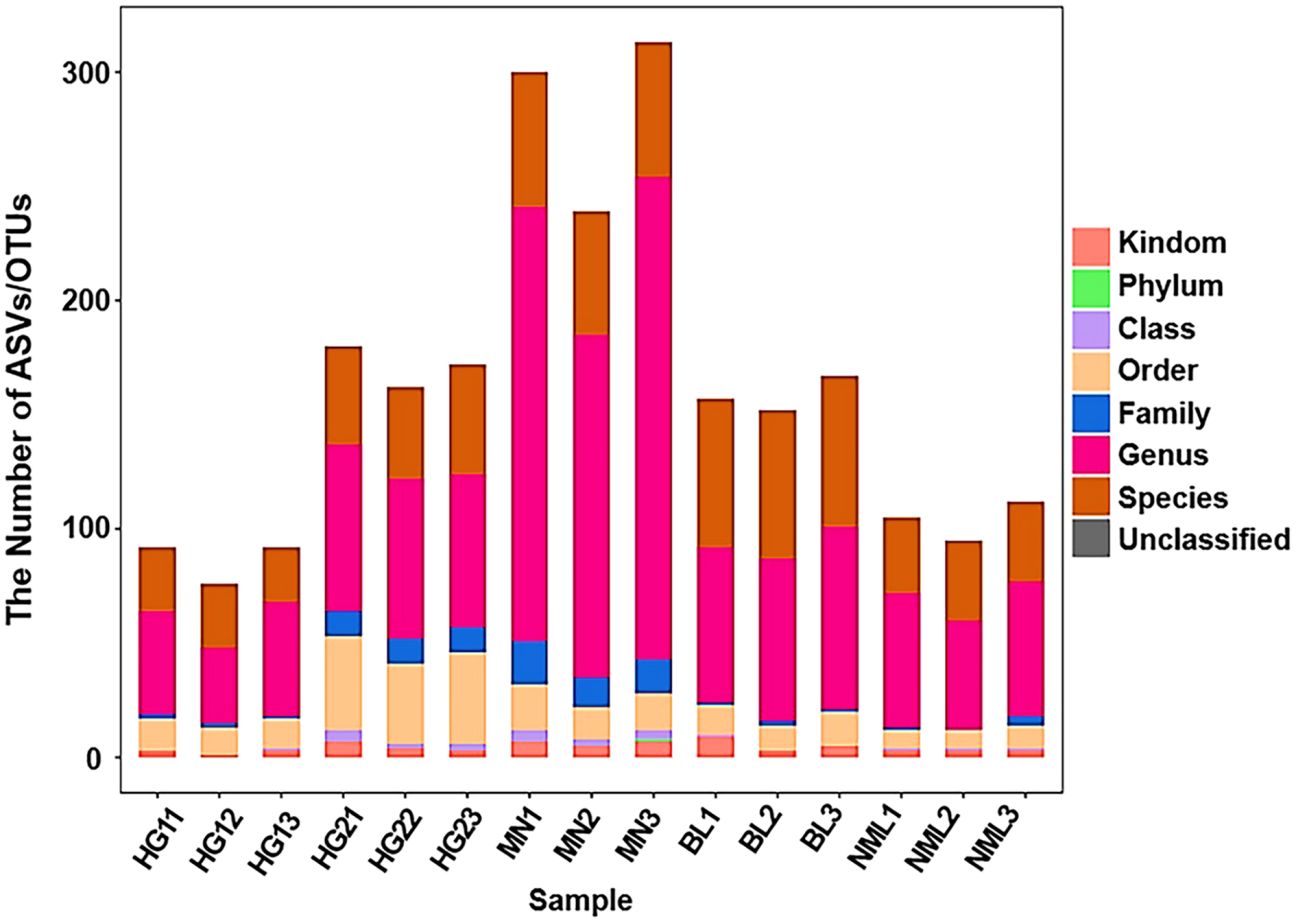
Statistical chart of taxonomic annotation results.

By sequencing the bacteria’s 16S rRNA gene’s V3–V4 region, generated a dataset of 946,672 raw sequences was filtered to achieve 792,642 high-quality sequences with an average length of 430 bp (shown in Table S1). The proportion of high-quality sequences to valid sequences was 90.22%. All valid sequences were clustered at a 97% similarity, with genera level copolymerizing into 1,274 OUT and species level copolymerizing to 682 OTU (shown in Table S2). As shown in Fig. 2, the abscissa coordinate was arranged according to the name of the fermented yogurt sample in different regions, and the ordinate coordinate was the ASV/OTU number of each sample, The OUT quantity of MN sample is the highest, and the HG sample for sale is the lowest.

### Classification of sample bacterial flora at the phylum and genus levels

**Figure 3:**
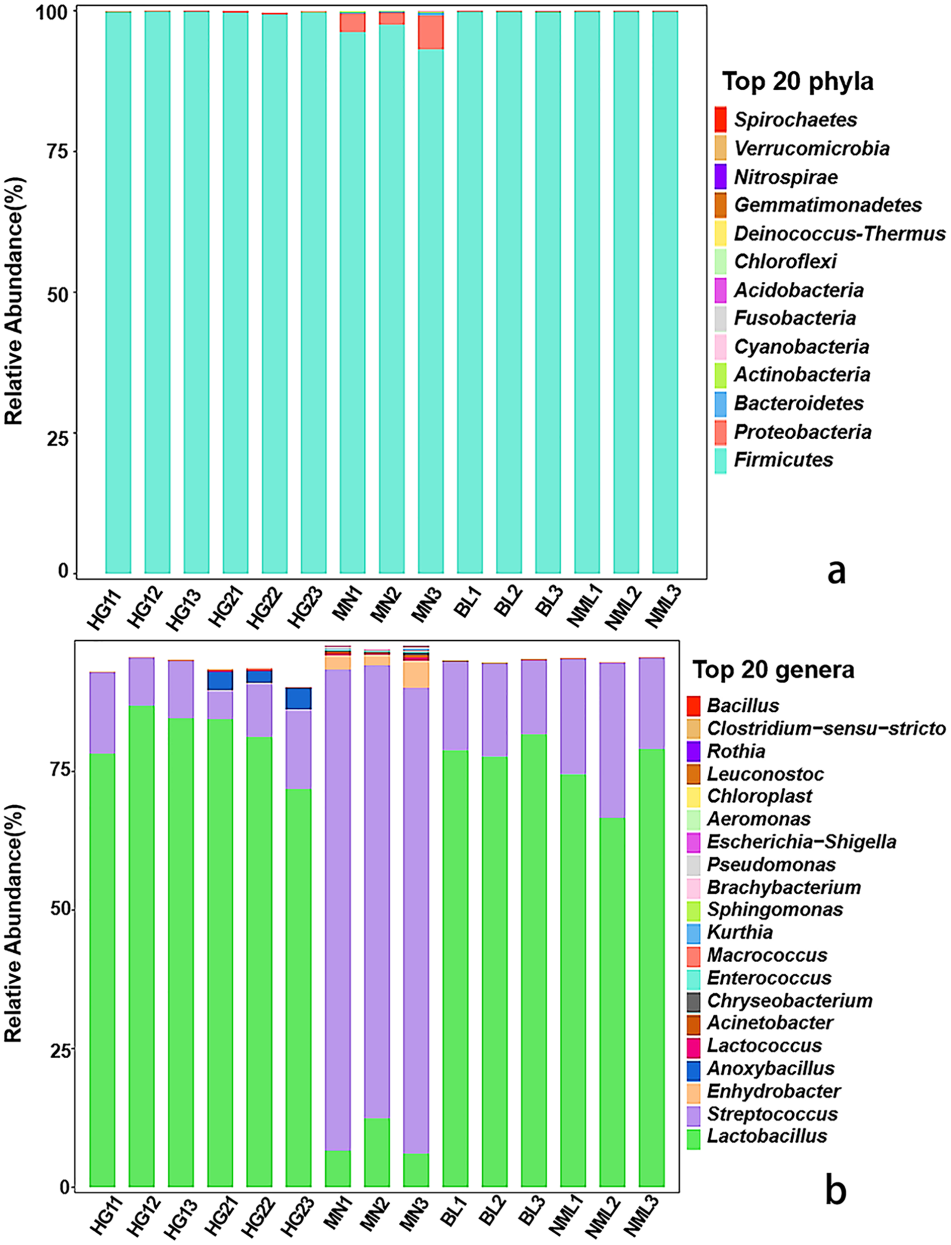
Relative abundances (percentage of sequences) of bacteria in fermented yak milk at phylum (A) and genus (B) level.

The distribution of each sample’s composition at various classification levels can be seen using the application analysis tool QIIME2 (2019.4). The results of the test showed that different samples were classified differently at the gate and genus levels, and different abundance values were shown for each phylum and genus. As can be seen from Fig. 3A, a total of 13 bacterial phyla were identified from 15 samples, *Firmicutes* as dominant phyla, the relative abundance of 15 samples ranged from 99.32% to 99.89%, and *Proteobacteria*, *Bacteroidetes*, *Cyanobacteria*, and *Actinobacteria* were low-abundance phyla. As shown in Fig. 3B, a total of 82 different bacterial strains and *unclassified* genera at the genus level were identified from 15 samples. Among them, *Lactobacillus* is a high-abundance genus, *streptococcus* is the second-most abundant genus, and it accounts for a relatively high proportion of MN samples. Low-abundance strains such as *Lactococcus*, *Enhydrobacter*, and *Pseudomonas* were also detected, and *Anoxybacillus* was detected only in sample HG2. The findings of our study were consistent with those of Jiang et al. (2019), who found that the fermented yak milk samples from the Aba Tibetan autonomous region of China varied in the abundance of microbiota at the genus level, with *Lactobacillus* being the most abundant genus in the majority of samples, ranging from 41.6% to 98.26%.

### Alpha diversity index

**Figure 4:**
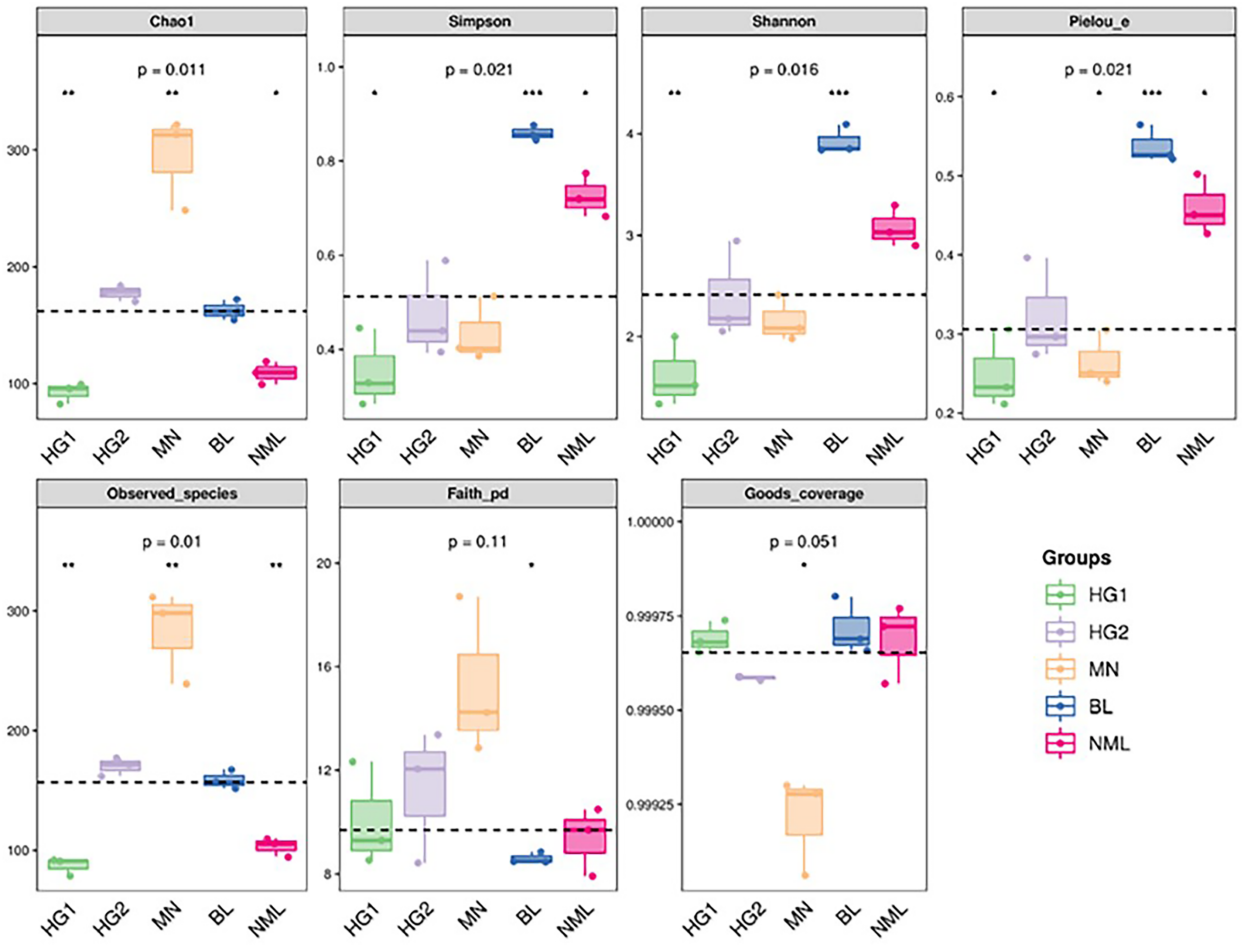
Grouped boxplot of the alpha diversity index.

The alpha diversity of microbial communities was estimated using the QIIME platform for a more comprehensive assessment of microbial community structure. Coverage was characterized by the Good’s coverage index, richness by Chao1 and Observed species index, diversity by Shannon and Simpson indexes, evolution-based diversity by Faith’s PD index, and uniformity by Pielou’s evenness index. The results were shown in Fig. 4, and the Good’s coverage rate of all samples was above 99.9%, indicating that most bacterial systemic types have been detected. From the Chao1 and Observed species indices, it suggested that the richness of MN samples is higher than that of other samples. The Shannon and Simpson, and Pielou’s evenness indices were higher for both the BL and NML samples, indicating that the diversity and uniformity of the two samples were superior to that of the others. The Faith’s PD index was similar across all samples, indicating they had similar evolutionary diversity. In summary, the diversity of bacterial flora varies from source to sample.

### Rarefaction curves

**Figure 5:**
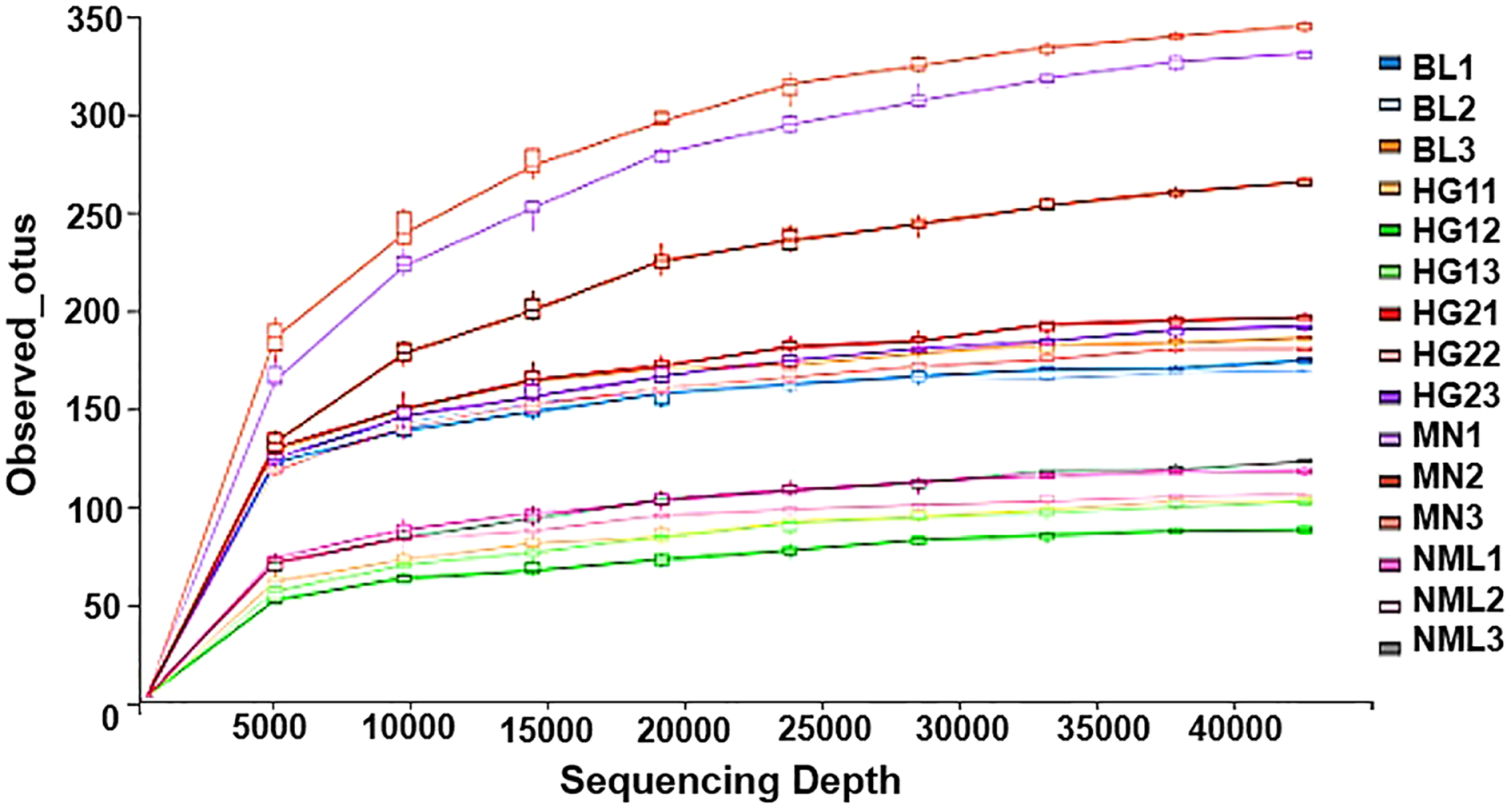
Rarefaction curves of alpha diversity index.

Rarefaction curves represent the situation with the increase of sequencing quantity, the species that may be detected will increase. It is widely used to judge whether sequencing has reached a certain depth (Sarr et al., 2019). The flattening degree of the curve reflects the influence of sequencing depth on the diversity of observed samples. A huge number of previously unknown novel ASVs or OTUs could not be detected by further increasing the sequencing depth, according to the flattening degree of the curve, which showed that the sequencing results were sufficient to reflect the variety of the existing samples. Otherwise, it indicated that alpha diversity was not close to saturation. As can be seen from Fig. 5, with the increase of sequencing amount, the OTUs of the sample gradually increased and then became stable, indicating that the sequencing depth was sufficient and basically covered all species in samples, and showing that the samples collected in this study could represent the species composition of fermented yak milk community in this region.

### Beta diversity analysis

Beta diversity, as the comparative analysis of the composition of microbial communities in different samples, it could reflect differences in diversity between different samples.

#### Principal coordinate (PCoA) analysis

**Figure 6:**
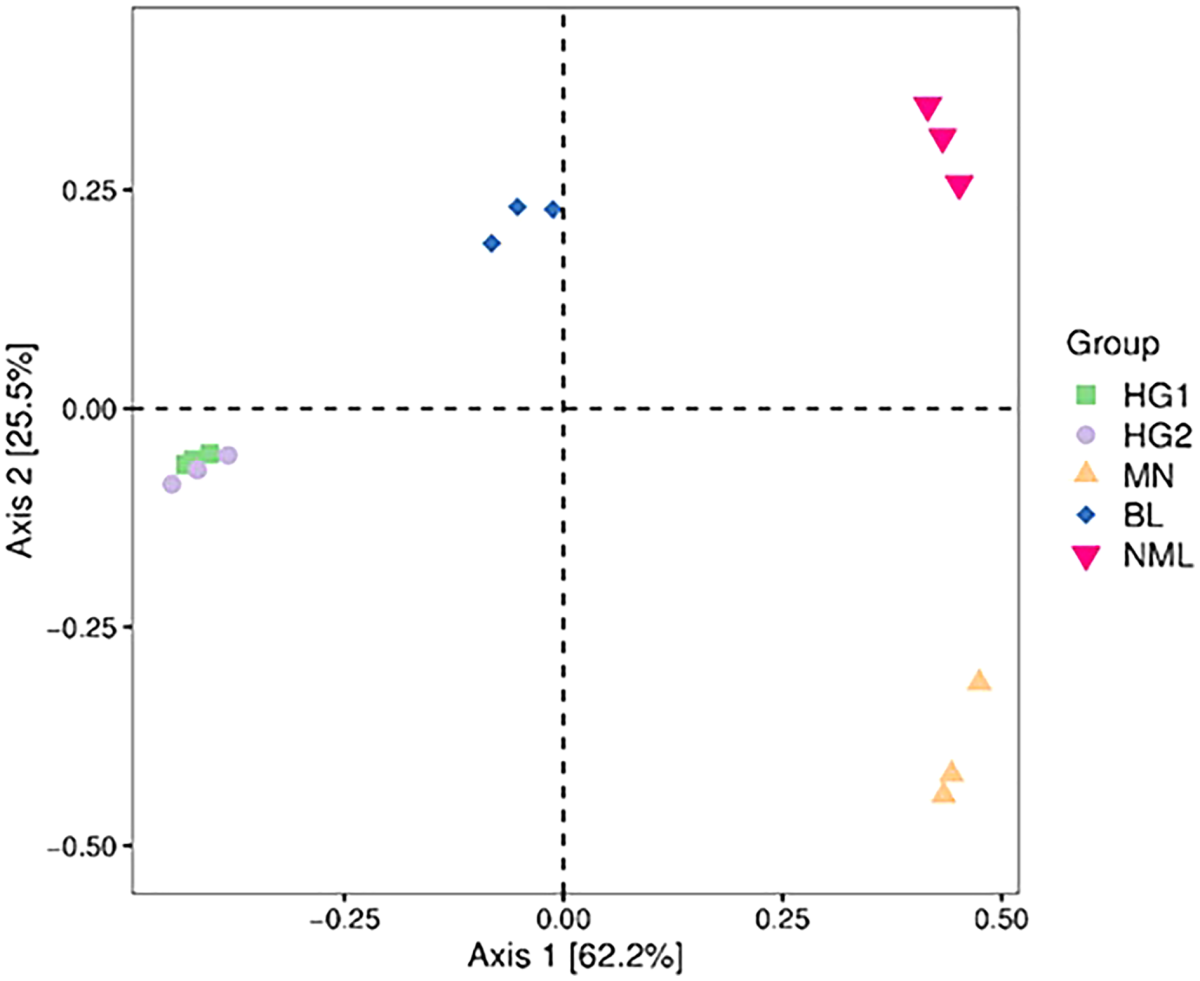
Two-dimensional sorting plot of samples for PCoA analysis.

PCoA combines clustering and principal component analysis methods to sort taxa with fewer principal coordinates and minimize information loss. Figure 6 depicts the PCoA analysis of 15 samples, with the distance between samples representing the similarity of the microbial communities of each traditional fermented yak milk. The closer the distance, the more similar the community composition of the samples in the corresponding dimensions. The percentages in the axis brackets represent the proportion of the sample difference data (distance matrix) that can be interpreted by the corresponding axis, which is 62.2% and 25.5%, respectively. From Fig. 6, it can be seen that the fermented yak milk communities in the same region overlap in the Axis 1 and Axis 2 dimensions, indicating that the similarity of fermented yak milk samples in the same region is high, and the HG1 and HG2 samples are similar in distance and overlap, which can indicate that the samples in these two regions have some bacteria OUT in common; while the samples in the other three regions are farther apart, indicating that their community composition is different.

#### Cluster analysis of sample flora structure

**Figure 7:**
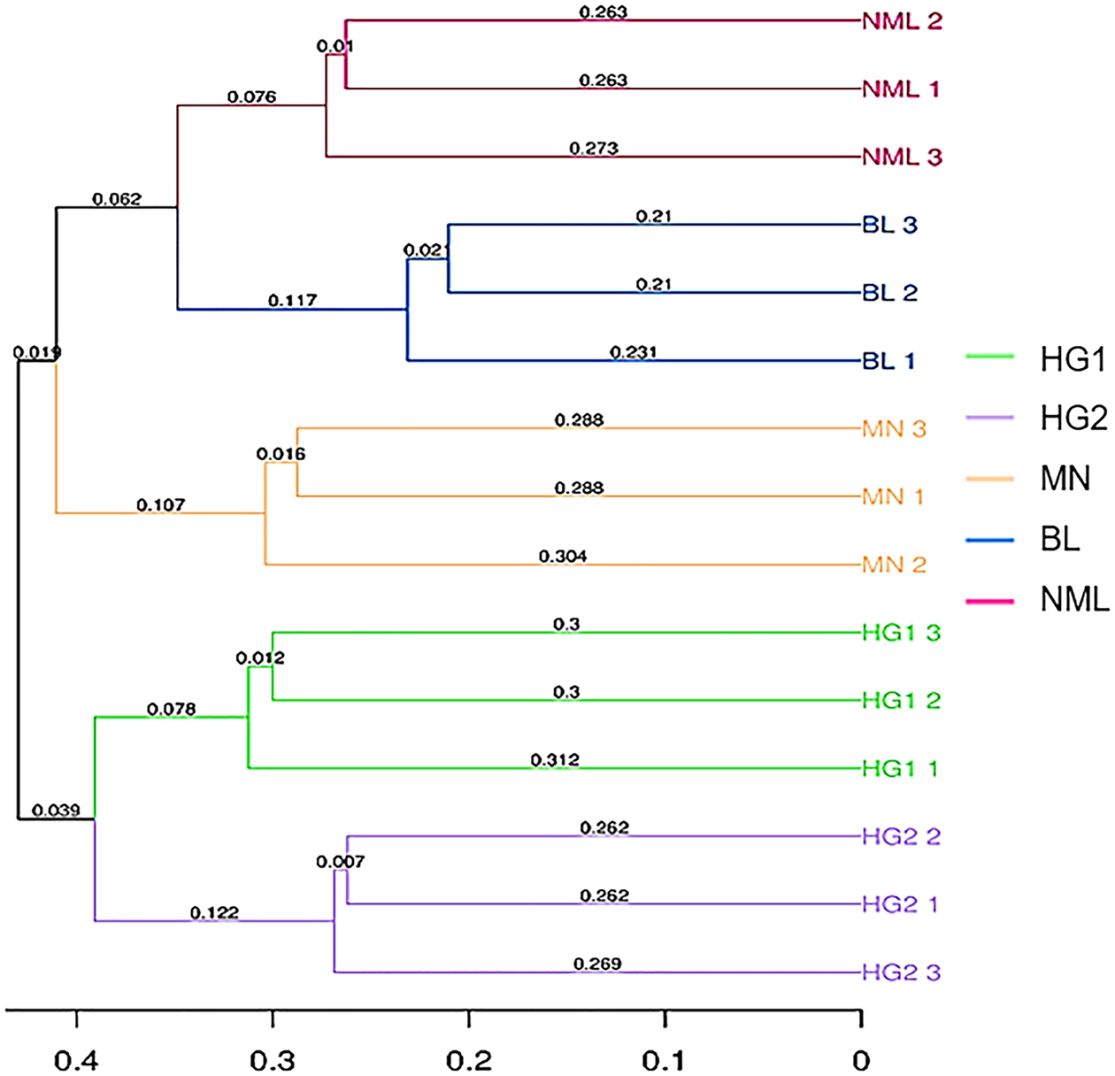
UPGMA clustering tree based on sample distance matrix.

Sample clustering refers to using arithmetic mean group pairs (UPGMA, Unweighted pair-group method with arithmetic means) to analyze the significant differences in samples in a particular evolutionary lineage (Zhang et al., 2018). The more similar the two samples, the shorter the branch length between the samples. From Fig. 7, it can be seen that 15 traditional fermented yak milk samples were clustered into two categories, and the NML, BL, and MN branches in the first category were small and the similarity was higher; the HG1, and HG2 branches were small, and the similarity was high, indicating that the difference in flora of fermented yogurt samples in the two regions was not significant. The NML and BL branches are smaller and more similarly clustered into one class, indicating that they have similar evolutionary lineages; MN is clustered separately and exhibits different microbial community characteristics compared to the samples in the Gannan region of Gansu Province. This is consistent with the results of the PCoA analysis.

### Heat map of species composition

**Figure 8:**
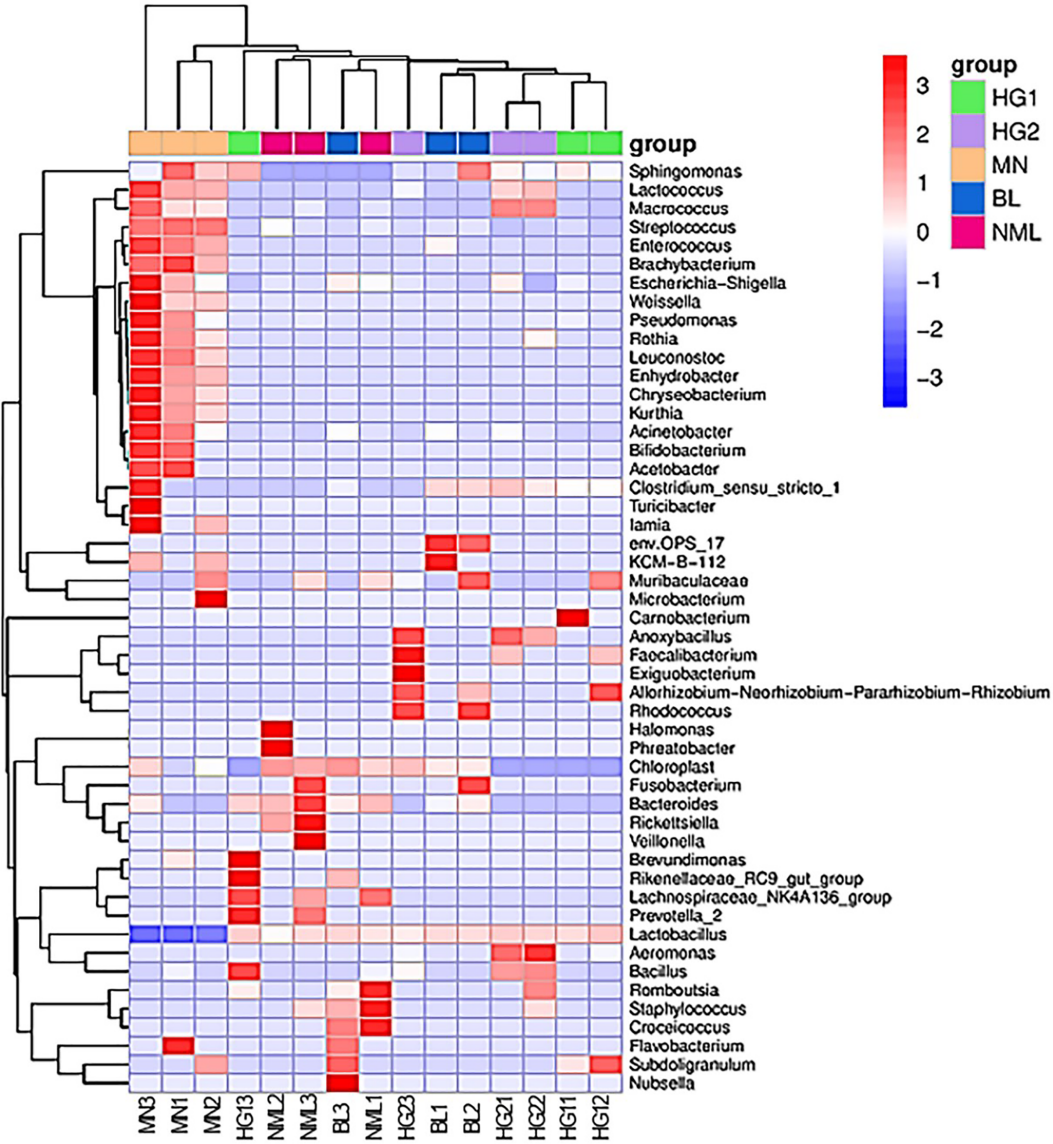
Heat map of bipolymer (genus level) species composition.

To study the species composition differences between fermented yogurts of yaks in different regions, heat maps were used for species composition analysis, and the abundance data of the top 50 genera with average abundance were plotted using genera-level taxa. As shown in Fig. 8, a double cluster heat map was plotted based on the correlation between five parts of traditional fermented yak milk samples. The red color block represented the genus abundances in the yogurt sample was higher than that of other samples, and the blue color block represents that the abundance of the genus in the yogurt sample was lower than that of other samples. It can be seen from Fig. 8 that there are differences in the diversity of bacteria in different types of samples. Among the samples of different types of traditional yak fermented dairy products, fermented yak milk MN3 and MN1 contain more bacteria. The contents of other *lactobacillus* were the least, indicating that the abundance of bacteria in each sample was different.

### PICRUSt function predictive analysis

**Figure 9:**
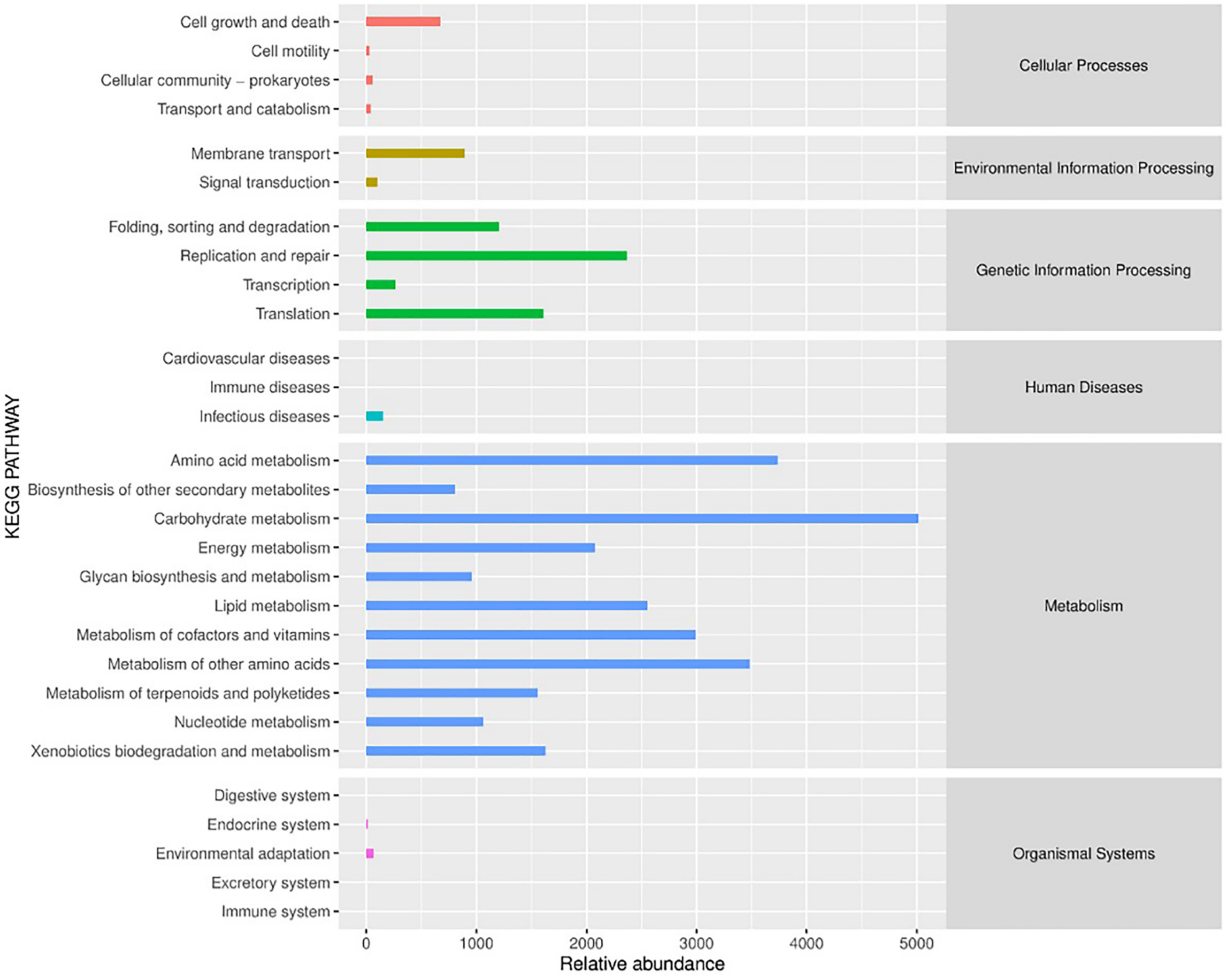
Histogram of KEGG metabolic pathway.

PICRUSt (Phylogenetic Investigation of Communities by Reconstruction of Unobserved States) is a software that predicts the functional abundance of a sample based on the abundance of marker gene sequences in the sample (Gavin et al., 2019). Here, the function refers to the gene family, such as KEGG ortholog gene, EC enzyme classification number, *etc*. In this study, PICRUSt software was applied to predict the gene functions of bacterial flora in different fermented yak milk samples. In addition to predicting gene functions, the predicted functional categories were annotated with reference to KEGG database. As can be seen from Fig. 9, the bacterial functional genes in KEGG database were Cellular Processes (2.4%), Environmental Information Processing (2.98%) and Genetic Information Processing (16.33%), Human Diseases (0.46%), Metabolism (77.6%) and Organismal Systems (0.23%) were mainly related to Metabolism. Each functional gene could be divided into several subclasses, a total of 29 subclasses, indicating that the abundance of bacterial metabolic genes in fermented yak milk was relatively high. In different subsets, it can be seen that there are different concentrations of different Amino acids in Different rumen (15.05%), Amino acid metabolism (11.21%), and metabolism of other Amino Acids (10.44%), Metabolism of Cofactors and nutrition (8.97%), Lipid Metabolism (7.66%), The genes related to metabolism (6.23%) were predominant among the predicted metabolic functional genes. Replication and repair (7.1%), Translation (4.83%), Folding, sorting and degradation (3.61%) were also present. These results indicate that many bacteria in traditional fermented yak milk contain abundant metabolic genes and can carry out a variety of growth and metabolic activities. The higher KEGG abundance, the stronger metabolic function of microorganisms. According to KEGG functional classification statistics, the metabolic function of microorganisms in fermented yak milk was extensive, and the destruction of normal bacterial community structure may hinder these metabolic pathways.

## Discussion

The Tibetan Plateau is rich with unique biological species resources, because of its particular geographical and climatic environment. Traditional fermented yak milk, with a history of over a thousand years contains abundant native lactic acid bacteria with special biological functions, which is a valuable and unique microbial germplasm resource under extreme environmental conditions. Among them, most lactic acid bacteria play an important probiotic role in the health of humans and animals, but some of them can cause diseases in humans and animals and are clinically meaningful pathogenic bacteria. Therefore, in order to make use of the beneficial microorganisms in the actual production and application, it is necessary to understand their characteristics comprehensively and correctly. Traditional fermented yak milk has many untapped, possibly advantageous bacteria as a conventionally fermented product. Exploring the microbial community structure has considerable assistance from high-throughput sequencing. This study aimed to provide a broad overview of the microbiota diversity of naturally fermented dairy products across a wide region of Gannan. Thus, we reanalyzed the High-throughput sequencing of 15 naturally fermented milk samples from different farmers in four villages from Xiahe County (3,500 m above sea level) in the Gannan Tibetan Autonomous Prefecture in Gannan.

In our analysis, 13 bacterial phyla were identified at the gate level from 15 samples, *Firmicutes* as dominant phylum, the relative abundance of 15 samples ranged from 99.32% to 99.89%, and *Proteobacteria*, *Oidetes*, *Cyanobacteria*, and *Actinobacteria* were low-abundance phyla, each sample exhibits a different abundance, and a low-abundance *Bacteroides* and *Cyanobacter* phyla were identified. These results were consistent with the findings of fermented camel milk in Tacheng Region of Xinjiang (Zhang et al., 2017b), fermented kefir in Zhaosu and Turks Counties in Xinjiang (Xu et al., 2014), and fermented goat dairy products in Yunnan (Liu et al., 2015a).

At the genus level, *Lactobacillus* is a high-abundance genus, *streptococcus* is the second-most abundant genus, and it accounts for a relatively high proportion of MN samples. Low-abundance strains such as *Lactococcus*, *Enhydrobacter* and *Pseudomonas* were also detected, and *Anoxybacillus* was detected only in sample HG2. Naturally fermented dairy products are frequently associated with *Lactobacillus* and *Streptococcus*, crucial to dairy product synthesis and fermentation (Bao et al., 2012a; Watanabe et al., 2008). Acid tolerance in the genus *Lactobacillus* is reported to be higher than in *streptococcus* and *Lactococcus* (Rogosa, Mitchell & Wiseman, 1951). During the fermentation of yak milk, as the acidity rises, a high proportion of *lactobacilli* are retained and enriched, so *Lactobacillus* becomes a highly abundant genus. In the study of Bao et al. (2012b), the high-throughput sequencing and PCR-DGGE were applied to determine the bacterial diversity in the acidic gruel from Inner Mongolia. Similar to the findings of this investigation, the results showed that *Lactobacillus* and *Acetobacter* had the highest relative contents. Other studies also have indicated that *Lactobacillus* had the potential to inhibit the other pathogens’ growth (Inoue et al., 2017). It is important to note that the samples we gathered also contained *Acinetobacter*, *Enhydrobacter*, *Anoxybacillus*, *Chryseobacterium*, *Macrococcus*, and *Aeromonas*. These might be connected to the traditional fermented yak milk’s open manufacturing setting (Bokulich et al., 2015; Aldrete-Tapia et al., 2014). In addition, the results of this study show that *Escherichia* was detected in all samples, and the proportion of non-sold samples was higher than that of sold samples. In addition, *Staphylococcus* was also detected in a few samples, so we speculate that the cooperative’s relatively standardized production effectively reduced the contamination degree of fermented yak milk. Therefore, herders should be encouraged to pay attention to hygiene throughout the production process, including milking and sterilizing all containers used, with the aim of producing traditional fermented yak milk that is both hygienic and of high quality.

Zhong et al. (2016) provided an overview of the bacterial microbiota biodiversity of 85 samples, previously collected across a wide region of China, Mongolia, and Russia. The most prevalent phyla shared across samples were *Firmicutes*, *Proteobacteria*, *Bacteroidetes*, and *Actinobacteria*, which together accounted for 99% of bacterial sequences. The predominant genera were *Lactobacillus*, *Lactococcus*, *Streptococcus*, *Acetobacter*, and *Acinetobacter*, which together corresponded to 96.63% of bacterial sequences. Further multivariate statistical analyses revealed significant differences in the microbiota structure across sample geographic origin and type. In our results, these phyla and genera of bacteria were also detected, but the abundance was different.

Based on Illumina MiSeq high-throughput technology, the diversity of bacterial flora in traditional fermented yak milk in Gannan was analyzed. The results indicated that there were certain differences in the structure of bacterial flora in traditional fermented yak milk samples from different sources. Liu et al. (2015c) investigated that the bacterial and fungal community diversity of 19 naturally fermented cow’s milk (NFCM) samples from local Mongolian families residing in Russia, which suggested that structural differences existed in the microbiota of NFCM. The difference in geographic environment may be an important factor influencing the microbial diversity of NFCM made by the Mongolians in Russia. It has been reported that the raw material from various environments influences the microbial changes in the product (Quigley et al., 2012). The microbiota of dairy fermentations can be influenced by seasonal variations in ingredients (Wullschleger et al., 2013). A study discovered an intriguing relationship between lipids and bacterial populations in milk from four different geographical locations (Asia, Africa, and North and South Europe) (Kumar et al., 2016). One such example is the negative relationship between the Lactobacillus genus and monounsaturated fatty acids (Damaceno et al., 2017). The proportion of monounsaturated fatty acids in yak milk increased with altitude (Cui et al., 2016). Overall, natural environmental and processing factors, such as altitude, temperature, oxygen concentration, raw material and manufacturing process, can influence their composition and microbiota more or less (Wu et al., 2009; Bokulich et al., 2015; Zhong et al., 2016; Ding et al., 2022). The fermented yak milk collected in this study came from four villages with similar environmental conditions, which can ensure that the yaks live in the same climate and eat the same forage variety. Moreover, the yaks in this area are genetically similar, so the yak genes are the same and the ingredients of raw milk are not different. Thus, we speculated that the manual technological process is the main reason, including the use of starter cultures and the fermentation process (such as pasteurization temperature and time), there is no uniform standard, causing the region bacterial community composition differences in fermented yak milk. People living on the plateau suffer from the stress of the harsh environment, such as low temperature, low oxygen, and strong ultraviolet rays all year. Moreover, the traditional customs of eating a single diet, drinking more alcohol and eating more meat have caused a variety of diseases. Lactic acid bacteria in traditional fermented yak milk have many probiotic functions, such as antioxidant, cholesterol-lowering, lipid-lowering, and other beneficial effects. Therefore, it is a feasible decision to use local high-quality microbial resources for microecological treatment to alleviate local common diseases, which needs to be further studied.

## Conclusions

Based on Illumina MiSeq high-throughput technology, the diversity of bacterial flora was analyzed on traditional fermented yak milk in Gannan. The results indicated that the fermented yak milk in Gannan had rich bacterial diversity, the dominant phylum was *Firmicutes*, the dominant genus was *Lactobacillus*, and different sources of fermented yak milk showed different abundance at the phylum level. Beta diversity analysis results suggested that the community similarity was high and there were similar evolutionary lineages. Based on KEGG functional classification, the microorganisms in traditional fermented yak milk have rich metabolic functions. Our results provided a theoretical basis for further exploring the microbial fauna in Gannan traditional fermented yak milk.

## Supplemental Information

10.7717/peerj.14733/supp-1 Supplemental Information 1 Statistical table of sequencing volume per sample.

10.7717/peerj.14733/supp-2 Supplemental Information 2 Statistical table of species taxonomy annotation results.

10.7717/peerj.14733/supp-3 Supplemental Information 3 BioProject: PRJNA881731.

10.7717/peerj.14733/supp-4 Supplemental Information 4 Object IDs and corresponding URLs.

10.7717/peerj.14733/supp-5 Supplemental Information 5 HG11 sample sequence.

10.7717/peerj.14733/supp-6 Supplemental Information 6 HG12 sample sequence.

10.7717/peerj.14733/supp-7 Supplemental Information 7 HG12 sample sequence.

10.7717/peerj.14733/supp-8 Supplemental Information 8 HG13 sample sequence.

10.7717/peerj.14733/supp-9 Supplemental Information 9 HG13 sample sequence.

10.7717/peerj.14733/supp-10 Supplemental Information 10 HG21 sample sequence.

10.7717/peerj.14733/supp-11 Supplemental Information 11 HG23 sample sequence.

10.7717/peerj.14733/supp-12 Supplemental Information 12 NML3 sample sequence.

10.7717/peerj.14733/supp-13 Supplemental Information 13 BL1 sample sequence.

10.7717/peerj.14733/supp-14 Supplemental Information 14 BL1 sample sequence.

10.7717/peerj.14733/supp-15 Supplemental Information 15 MN2 sample sequence.

10.7717/peerj.14733/supp-16 Supplemental Information 16 HG22 sample sequence.

10.7717/peerj.14733/supp-17 Supplemental Information 17 HG22 sample sequence.

10.7717/peerj.14733/supp-18 Supplemental Information 18 HG21 sample sequence.

10.7717/peerj.14733/supp-19 Supplemental Information 19 NML2 sample sequence.

10.7717/peerj.14733/supp-20 Supplemental Information 20 NML1 sample sequence.

10.7717/peerj.14733/supp-21 Supplemental Information 21 BL3 sample sequence.

10.7717/peerj.14733/supp-22 Supplemental Information 22 NML3 sample sequence.

10.7717/peerj.14733/supp-23 Supplemental Information 23 MN1 sample sequence.

10.7717/peerj.14733/supp-24 Supplemental Information 24 MN1sample sequence.

10.7717/peerj.14733/supp-25 Supplemental Information 25 MN3 sample sequence.

10.7717/peerj.14733/supp-26 Supplemental Information 26 HG11 sample sequence.

10.7717/peerj.14733/supp-27 Supplemental Information 27 HG23 sample sequence.

10.7717/peerj.14733/supp-28 Supplemental Information 28 MN3 sample sequence.

10.7717/peerj.14733/supp-29 Supplemental Information 29 MN2 sample sequence.

10.7717/peerj.14733/supp-30 Supplemental Information 30 BL2 sample sequence.

10.7717/peerj.14733/supp-31 Supplemental Information 31 BL3 sample sequence.

10.7717/peerj.14733/supp-32 Supplemental Information 32 BL2 sample sequence.

10.7717/peerj.14733/supp-33 Supplemental Information 33 NML2 sample sequence.

10.7717/peerj.14733/supp-34 Supplemental Information 34 NML1 sample sequence.

## Additional Information and Declarations

### Data Availability

The following information was supplied regarding data availability: Data is available at NCBI, Sequence Read Archive (SRA): PRJNA881731: SRR21661706, SRR21661705, SRR21661699, SRR2166198, SRR21661697, SRR21661696, SRR21661695, SRR21661694, SRR21661693, SRR21661692, SRR21661704, SRR21661703, SRR21661702, SRR21661701, SRR21661700.
