# Supplementary material for: Research on bacterial community characteristics of traditional fermented yak milk in the Tibetan Plateau based on high-throughput sequencing
Source: PeerJ. 2023 Jan 25;11:e14733. doi: 10.7717/peerj.14733 (PMC9884033; doi:10.7717/peerj.14733)
Supplement: Supplemental Information 1 [file peerj-11-14733-s001.docx]

| Sample ID | Input | Filtered | Denoised | Merged | Non-chimeric | Non-singleton |
| --- | --- | --- | --- | --- | --- | --- |
| HG11 | 65389 | 60754 | 60509 | 60333 | 56131 | 56127 |
| HG12 | 65557 | 61112 | 60923 | 60765 | 58416 | 58409 |
| HG13 | 65628 | 61120 | 60914 | 60794 | 58296 | 58291 |
| HG21 | 62388 | 57026 | 56756 | 56560 | 53928 | 53896 |
| HG22 | 60442 | 56199 | 55937 | 55775 | 53011 | 52969 |
| HG23 | 65814 | 61476 | 61204 | 60944 | 54705 | 54661 |
| MN1 | 63386 | 59671 | 59381 | 59157 | 54162 | 54104 |
| MN2 | 62798 | 58539 | 58190 | 57988 | 51103 | 51055 |
| MN3 | 63986 | 59953 | 59606 | 59285 | 54997 | 54942 |
| BL1 | 64255 | 60098 | 59846 | 59684 | 53831 | 53806 |
| BL2 | 59673 | 55725 | 55478 | 55361 | 48617 | 48606 |
| BL3 | 61577 | 57186 | 56934 | 56826 | 51174 | 51160 |
| NML1 | 63669 | 59318 | 59074 | 58827 | 49650 | 49640 |
| NML2 | 58924 | 55056 | 54836 | 54647 | 44898 | 44891 |
| NML3 | 63186 | 59204 | 58962 | 58801 | 49723 | 49706 |
| total | 946672 | 882437 | 878550 | 875747 | 792642 | 792263 |

Table 1 Statistical table of sequencing volume per sample
