# Supplementary material for: Research on bacterial community characteristics of traditional fermented yak milk in the Tibetan Plateau based on high-throughput sequencing
Source: PeerJ. 2023 Jan 25;11:e14733. doi: 10.7717/peerj.14733 (PMC9884033; doi:10.7717/peerj.14733)
Supplement: Supplemental Information 2 [file peerj-11-14733-s002.docx]

Table 2 Statistical table of species taxonomy annotation results

| ID | domain | phylum | class | order | family | genus | species | unclassified |
| --- | --- | --- | --- | --- | --- | --- | --- | --- |
| HG11 | 3 | 0 | 0 | 14 | 2 | 45 | 28 | 0 |
| HG12 | 1 | 0 | 0 | 12 | 2 | 33 | 28 | 0 |
| HG13 | 3 | 0 | 1 | 13 | 1 | 50 | 24 | 0 |
| HG21 | 7 | 0 | 5 | 41 | 11 | 73 | 43 | 0 |
| HG22 | 4 | 0 | 2 | 35 | 11 | 70 | 40 | 0 |
| HG23 | 3 | 0 | 3 | 40 | 11 | 67 | 48 | 0 |
| MN1 | 7 | 0 | 5 | 20 | 19 | 190 | 59 | 0 |
| MN2 | 5 | 0 | 3 | 14 | 13 | 150 | 54 | 0 |
| MN3 | 7 | 1 | 4 | 16 | 15 | 211 | 59 | 0 |
| BL1 | 9 | 0 | 1 | 13 | 1 | 68 | 65 | 0 |
| BL2 | 3 | 0 | 0 | 11 | 2 | 71 | 65 | 0 |
| BL3 | 5 | 0 | 0 | 15 | 1 | 80 | 66 | 0 |
| NML1 | 3 | 0 | 1 | 8 | 1 | 59 | 33 | 0 |
| NML2 | 3 | 0 | 1 | 8 | 0 | 48 | 35 | 0 |
| NML3 | 3 | 0 | 1 | 10 | 4 | 59 | 35 | 0 |
| total | 66 | 1 | 27 | 270 | 94 | 1274 | 682 | 0 |
